# A Bond-Based Peridynamic Model with Matrix Plasticity for Impact Damage Analysis of Composite Materials

**DOI:** 10.3390/ma16072884

**Published:** 2023-04-04

**Authors:** Mingwei Sun, Lisheng Liu, Hai Mei, Xin Lai, Xiang Liu, Jing Zhang

**Affiliations:** 1 Hubei Key Laboratory of Theory and Application of Advanced Materials Mechanics, Wuhan University of Technology, Wuhan 430070, China; 2 Department of Engineering Structure and Mechanics, Wuhan University of Technology, Wuhan 430070, China

**Keywords:** bond-based peridynamic theory, composite materials, plastic hardening, impact, damage

## Abstract

The prediction of damage and failure to fiber-reinforced polymer composites in extreme environments, particularly when subjected to impact loading, is a crucial issue for the application and design of protective structures. In this paper, based on the prototype microelastic brittle (PMB) model and the LaRC05 composite materials failure model, we proposed a bond-based peridynamic (BB-PD) model with the introduction of plastic hardening of the resin matrix for fiber-reinforced polymer composites. The PD constitutive relationships of the matrix bond and interlayer bond under compressive loading are considered to include two stages of linear elasticity and plastic hardening, according to the stress–strain relationship of the resin matrix in the LaRC05 failure model. The proposed PD model is employed to simulate the damage behaviors of laminated composites subjected to impact loading. The corresponding ballistic impact tests of composite laminates were carried out to observe their damage behaviors. The PD prediction results are in good agreement with the ballistic experimental results, which can verify the correctness and accuracy of the PD model developed in this study in describing the impact damage behaviors of composite materials. In addition, the characteristics and degree of damage in composite laminates are analyzed and discussed based on this PD model. The difference in the impact resistance of composite laminates with different stacking sequences is also studied using the numerical simulation results.

## 1. Introduction

Fiber-reinforced composite materials are extensively applied in aviation, shipbuilding, military equipment, and automobile manufacturing due to their wonderful mechanical properties, such as high strength, modulus, fatigue resistance, and impact resistance. However, composite materials under extreme conditions, especially when subjected to impact loading, will inevitably appear damaged and fracture during their service life. Therefore, the damage prediction and analysis of composite materials is a critical issue when designing and using protective structures made from them.

Ballistic impact tests can be used to directly observe and study the complex damage phenomenon of composite materials. Gower et al. [1] researched the dynamic response and failure mechanism of aramid-laminated composites by conducting ballistic impact tests under projectiles with different geometric shapes. The experiment performed by Naik et al. [2] investigated the impact-induced damage of E-glass/epoxy thick laminated composites. Reddy et al. [3] conducted impact experiments on E-glass fiber-reinforced polymer composites and analyzed the effect of material parameters on the absorbed energy. Yang et al. [4] analyzed the mechanism of impact damage in composite materials using ballistic impact tests and revealed a correlation between the energy absorbed by the laminate and the residual velocity of the projectile. Although experimental observation and analysis is a well-established method to research the damage behavior and mechanism of composite materials, it has the disadvantages of high cost, low efficiency, and a long experimental period.

In order to more systematically investigate and describe the damage and fracture behaviors of fiber-reinforced composite materials, many theoretical failure models have been proposed. The first type of failure model is the fracture criterion approach, which mainly uses classical material strength theory and focuses on the ultimate fracture limit of composite materials. The Hoffman criterion [5] is applicable to the failure prediction of composite laminates with different tensile and compressive properties. Hashin et al. [6] established a fatigue failure criterion for composite materials under plane stress conditions considering the fiber and matrix fracture. The Tsai–Wu criterion [7] allows for the tensile and compressive strength of composite laminates to be inconsistent, but the strength interaction term in the theory must be determined by the biaxial test. The second type of failure model is the continuum damage mechanical (CDM) approach, which is characterized by considering the attenuation of the elastic modulus of the material. Pinho et al. [8] introduced a 3D failure criterion in order to describe the different forms of damage in composite laminates. Donadon et al. [9] built a full three-dimensional failure model to predict the damage of composite structures under multi-axial loads. This model can distinguish the damage that accumulates under different loading conditions (such as compression and tension). The third type of failure model is the plastic approach, which considers the non-linear mechanical properties of materials as plasticity. Vogler et al. [10] developed a novel transverse-isotropic elastoplastic constitutive model for unidirectional fiber-reinforced polymers. Batra et al. [11] considered the elastoplastic deformation of the polymer matrix and used the Hashin failure criterion to predict the failure of the composite. The plastic approach is widely used to study the damage and failure of composite materials because the matrix materials (such as polymers or metals) usually exhibit plastic behavior.

In addition to the failure models described above, a series of failure models for fiber-reinforced composites have been proposed by researchers from NASA. The first proposed failure model is called the LaRC03 model [12], which is based on the assumption of plane stress and provides the corresponding failure criteria for the matrix and fiber. Subsequently, the LaRC04 failure model [13] was proposed, which modified and supplemented the previously established LaRC03 model and made it suitable for the general 3D loading of composite materials and cases involving in-plane non-linear shear. The failure model of composite materials includes as much of the failure process and physical phenomena observed in the experiments as possible; the LaRC05 failure model [14] was proposed based on the previous LaRC03 and LaRC04 models. The model directly considers the non-linear responses in the shear, transverse and thickness directions, as measured by the experiments.

The LaRC05 failure model divides the failure modes of composite materials into three types, namely matrix cracking, fiber kinking failure, and fiber tensile fracture, and establishes the failure criteria corresponding to these three failure modes. In the matrix failure, the failure mechanism is a brittle fracture, and the constitutive equation is linearly elastic when the resin is subjected to a tensile load. When subjected to shear and compression loads, the failure mechanism of the resin is yield fracture; that is, the resin first shows linear elastic behavior, then shows plastic hardening behavior until the applied load exceeds the shear or compression strength of the resin, at which point the resin will fracture. Regarding fiber kinking failure, this model believes that the reason for its occurrence is as follows: when the composite laminates are under axial compression loading, the high shear stress caused by the failure between adjacent layers cracks the matrix between the fibers, and the matrix splitting promotes the further bending of the fibers, which in turn leads to more matrix cracking. As a result of the combination of bending and compressive stresses, the bent fiber eventually breaks at both ends and forms a kink zone. Fiber kinking failure can be determined using a failure index equation; when the index is greater than 1, fiber kink failure occurs. For fiber tensile failure, the authors believe that the results predicted using the classical maximum stress damage criterion are in good agreement with the experimental results and therefore predict the fiber tensile fracture based on this criterion. The LaRC05 failure model contains many physical phenomena related to the failure process of fiber-reinforced composites under three-dimensional stress states, so the failure prediction based on this model fits well with the experimental results.

Based on the strength criteria and failure models for composite materials, the finite element method (FEM) has been applied to the study of the damage and fracture of composite materials under impact loading [15,16,17,18,19]. However, FEM is mathematically difficult to deal with problems of discontinuity, such as damage and fracture, because it requires the deformation and stress within the object to remain continuous. The use of FEM to study damage and fracture problems may require remeshing and a prior knowledge of crack propagation path [20,21,22].

In order to avoid the difficulties of treating discontinuity problems such as damage and fracture to materials in terms of continuum mechanics, Dr. Silling developed a non-local theory based on the idea of non-local action, called peridynamic (PD) theory [23,24,25]. Unlike the FEM, PD theory uses spatial integral equations that can be applied to discontinuities to reformulate the basic equations of motion in continuum mechanics and allows for the emergence of discontinuous behaviors in objects. Therefore, the peridynamic theory is unique in analyzing and predicting the crack initiation and damage evolution of materials.

In recent years, the peridynamics theory has widely been employed to model the damage and fracture of composite materials. Xu et al. [26,27] used a bond-based PD model to predict the forms of damage, including delamination and matrix fracture, in composite laminates under bi-axial and low-velocity impact loading. An explicit model that distinguishes the different properties and volume fractions of fiber and matrix is employed by Kilic et al. [28] for the prediction of damage and failure in composite materials subjected to quasi-static loadings. Oterkus et al. [21] put forth a new approach based on a merger of the peridynamic theory and finite element method and used it to predict the damage to centrally slotted, curved laminated composites. Oterkus and Madenci [29] developed a bond-based PD model including the PD fiber, matrix, normal, and shear bonds of fiber-reinforced composite materials to simulate the damage evolution of laminated composites under static loading. However, there are two limitations in this model: the fiber directions cannot be chosen arbitrarily, and the micromodulus is not continuously changing. A new bond-based peridynamic model considering arbitrary laminate layups of fiber-reinforced composite materials was proposed by Hu et al. [30,31,32] in order to remove the restriction of a specific fiber direction. Zhou et al. [33] put forth a bond-based peridynamic model in which the bond parameter is continuous and used this to research the dynamic fracture behaviors and cracking velocity of laminated composites. Combining the PMB model and Kelvin–Voigt model, Sun and Huang [34] built a rate-dependent peridynamic model of composite materials and performed simulations of the impact-induced damage behaviors of laminated composites. Diyaroglu et al. [35] introduced a peridynamic model of composite laminates to study the damage and fracture of composite materials under explosive loading. Gao and Oterkus [36] predicted the damage to composite laminates under fire scenarios with a coupled thermo–fluid–mechanical peridynamic model. Ren et al. [37] conducted damage and resistive force research focusing on unidirectional laminated composites subjected to dynamic loading using the FEM-based PD model. Basoglu et al. [38] proposed a bond-based PD model that is suitable for simulating the toughening process of composite laminates and used it to analyze the effect of aggregated microcracks on the toughening of the resin matrix. The above studies have made remarkable contributions to the prediction of failure and revealed the mechanisms of damage and fracture of composite materials under static and dynamic loading from different angles of view, which enables people to obtain a prior understanding of the mechanical properties of composite materials when using and designing them.

Although the peridynamic theory has been effectively employed in composite failure prediction, the current PD model requires the introduction of plastic behaviors, which is essential when describing impact damage in composite materials [39]. The previous PD models are limited by their linear elasticity, which can only be applied to describe the brittle fracture behaviors of polymer composites. According to the LaRC05 failure model proposed by Pinho et al. [14], the resin matrix of composite materials shows obvious plastic behavior during the failure process under compression loading: the linear elastic stage occurs first, then the plastic hardening stage, and finally, fracture occurs. Therefore, an accurate description of the plastic failure behaviors of the resin matrix should be applied to the PD model of composite materials.

In this study, a bond-based PD model that considers the plastic hardening stage of the resin matrix under compression loading for composite materials is established based on the LaRC05 model [14] and the PMB model [24]. In the proposed PD model, the different mechanical behaviors that are subjected to the tensile and compressive loading of the resin matrix are considered. Furthermore, the corresponding model parameters are derived according to the peridynamic theory and continuum mechanics theory. In addition, a pairwise-force updating algorithm is presented to determine the true bond force when plastic deformation occurs. Using the proposed model and the numerical techniques, we simulated the impact-induced damage process of laminated composites and studied the corresponding failure characteristics and mechanisms under different conditions, including different impact velocities and stacking sequences.

## 2. Peridynamic Modeling for Composite Materials

### 2.1. Bond-Based Peridynamic Theory

**Figure 1:**
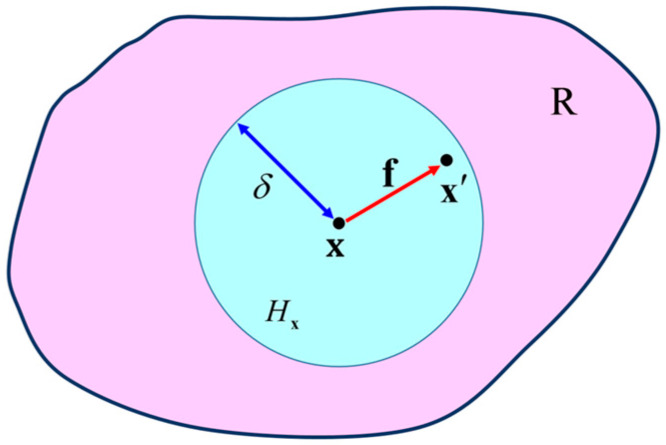
Schematic of peridynamic interactions.

The PD theory assumes that in a reference configuration R, each material point **x** owns an interaction domain *H***_x_** with a radius of *δ* called the horizon, as shown in Figure 1. The interaction between material point **x** and any material point **x′** located within the interaction domain *H***_x_** is characterized by a vector function **f**, which is called the pairwise force function (also called the bond force). **f** is given as follows

(1) $$f(\eta ,\xi )=f(\eta ,\xi )\frac{\eta +\xi }{\left\|\eta +\xi \right\|}.$$

**Figure 2:**
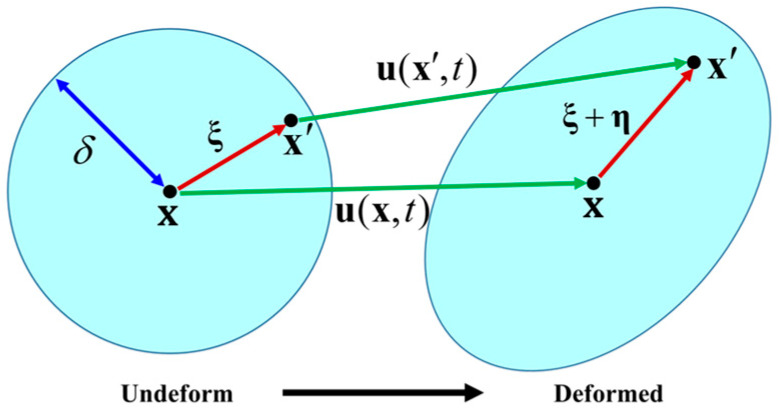
Initial and deformed configuration in peridynamics.

The peridynamics equation of motion for material point **x** at any instant of time *t* in the reference configuration R can be expressed as [23]

(2) $$\rho \left(x\right)\ddot{u}\left(x,t\right)=\int_{H}f(\eta ,\xi )dV^{\prime }+b\left(x,t\right),$$

where *ρ* is the mass density of the material point **x**; **ü** denotes the displacement vector field of the material point **x** at time *t*; and **b** is the prescribed body force density. *H***_x_** is the neighborhood of the material point **x**, which is characterized by the horizon *δ*. $dV^{\prime }$ represents the volume element of the object, in other words, $V^{\prime }$ denotes the volume possessed by the material point **x′.** The bond force **f** is a function of the relative position vector **ξ** in the reference configuration and the relative displacement vector **η** in the current configuration. As shown in Figure 2, vectors **ξ** and **η** are given as

(3) $$\xi =x^{\prime }-x,\eta =u\left(x^{\prime },t\right)-u\left(x,t\right),$$

Note that the existence of the solutions to Equations (2) and (3) was proved by Silling [23], the founder of PD theory. In addition, more mathematical information about the two-dimensional systems included in the above equations can be found in the literature [40,41].

The relative elongation between material point **x** and **x′** is defined as the bond stretch *s*, which can be expressed as

(4) $$s=\frac{\left\|\eta +\xi \right\|-\left\|\xi \right\|}{\left\|\xi \right\|}.$$

In the bond-based PD theory, the bond force **f** is linearly related to the bond stretch *s*, which is written as

(5) $$f=c\cdot s\frac{\eta +\xi }{\left\|\eta +\xi \right\|},$$

where *c* is the PD micro-modulus (bond parameter), which contains information about the constitutive relation of the material; When the material point is not in the horizon of material point **x**, the bond force between the two material points is zero. For isotropic linear-elastic materials, *c* = 18 *K/πδ*^4^, where *K* is the bulk modulus of the material.

The bond breaks once the bond stretch *s* exceeds its critical value *s*_0_. Thereafter, the interaction between material points **x** and **x′** disappears permanently, and the corresponding bond force remains at zero. For the PMB material, the critical stretch *s*_0_ depends on the critical energy release rate *G*_0_, given by [23]

(6) $$s_{0}=\sqrt{\frac{5G_{0}}{9K\delta }}.$$

In order to indicate the presence or breakage of the PD bond, a function *µ* is taken in the following form:

(7) $$\mu \left(x,t,\xi \right)=\left\{\begin{array}{l} 1\;\mathrm{if}\;s\left(t^{\prime },\xi \right)<s_{0}\;\mathrm{for}\;\mathrm{all}\;0\leq t^{\prime }\leq t\\ 0\;\mathrm{otherwise} \end{array}\right..$$

The continuous break of the bonds between material points forms a crack. Based on the number of broken bonds in the interaction domain of a material point **x**, the local damage at **x** is defined as

(8) $$\varphi \left(x,t\right)=1-\frac{\int_{H}\mu \left(x,t,\xi \right)dV^{\prime }}{\int_{H}dV^{\prime }}.$$

When the damage value *φ* is 0, there is no damage at the material point **x**, and all the bonds are intact in its interaction domain. In addition, when all bonds initially connected to point **x** are broken, the damage at point **x** reaches a maximum of 1.

### 2.2. PD Modeling of a Lamina

**Figure 3:**
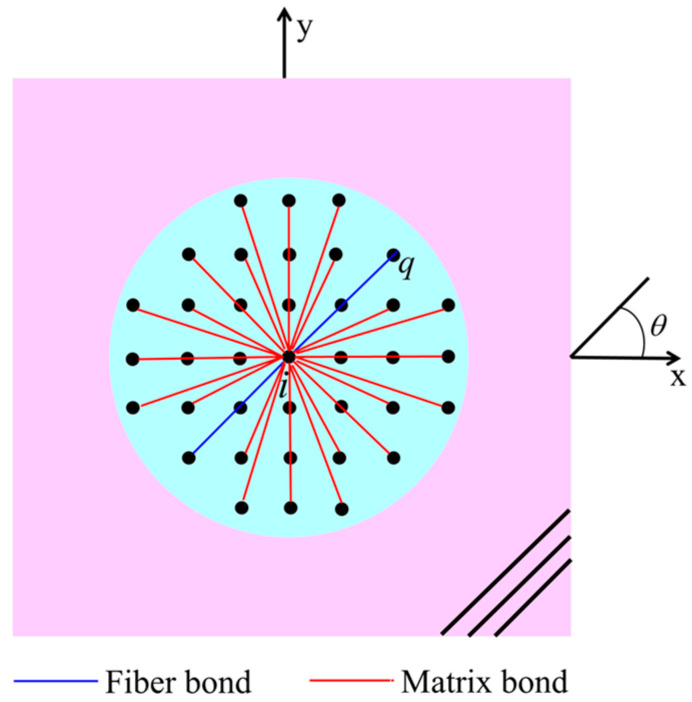
Two kinds of PD bonds in a lamina.

When using the PD theory to model and study isotropic materials, the mechanical properties of the bonds along different directions are exactly the same. For a composite lamina with anisotropy, the size of the interactions within the material depends on the bond directions. Therefore, this significant directional dependence has to be considered when modeling a composite lamina with a fiber direction of *θ* based on the peridynamics. The means to achieve this PD modeling requirement is to introduce two types of bonds with different properties (fiber bond and matrix bond), as shown in Figure 3. The fiber bond describes the interaction between two material points along the fiber orientation, while the matrix bond characterizes the interaction along any orientation within a lamina, including the fiber orientation [29]. The peridynamics parameters corresponding to the fiber bond and matrix bond are *c_f_* and *c_m_*, respectively.

#### 2.2.1. PD Model of the Fiber Bond

According to the fiber failure criteria in the LaRC05 model [14], the stress–strain relationship of the fiber is regarded as linearly elastic, and the fiber will fracture when its deformation exceeds the elastic limit. The prediction of fiber failure given by the classical maximum-stress criterion is in good agreement with the experimental results of fiber fracture. Therefore, the constitutive relation of the fiber bond will use the PMB model [24].

**Figure 4:**
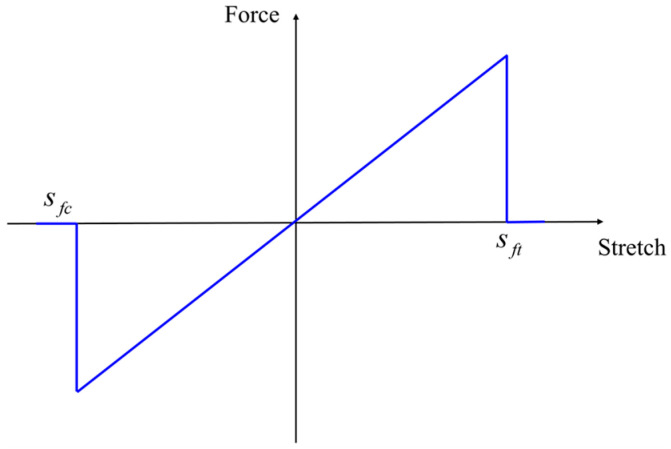
PD constitutive relation of the fiber bond.

The PD constitutive model of the fiber bond is shown in Figure 4. Following the PMB model, the constitutive relation of the fiber bond is written as

(9) $$f\left(\eta ,\xi \right)=\left\{\begin{array}{ll} c_{f}s\left(\eta ,\xi \right) & -s_{fc}\leq s\left(\eta ,\xi \right)\leq s_{ft}\\ 0 & \;\mathrm{otherwise} \end{array}\right.,$$

Note that the PD constant *c_m_* of the matrix bond along the fiber direction is ignored, as the strength of the fiber is much greater than that of the matrix. The parameters *s_ft_* and *s_fc_* are the critical stretches of the fiber bond under tensile and compressive conditions, respectively, and can be expressed as [23].

(10) $$s_{ft}=-s_{fc}=\sqrt{\frac{10G_{f}}{c_{f}\pi \delta^{5}}},$$

where *G_f_* is the critical energy-release rate of fiber material.

#### 2.2.2. PD Model of the Matrix Bond

**Figure 5:**
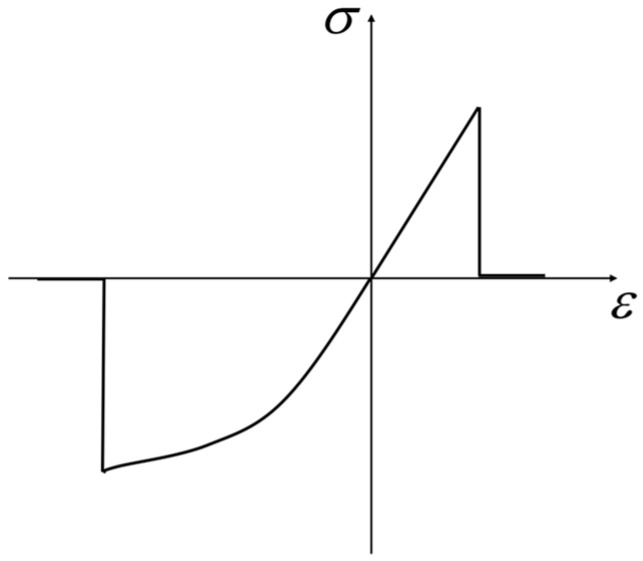
ε-σ relation of the resin matrix.

In previous studies, the PD constitutive model of the matrix bond is also linear elastic, similar to that of the fiber bond. The critical stretches of the matrix bond are specified as that of the matrix material critical stretch, *s_mt_* and *s_mc_*, under tensile and compressive conditions, respectively [42]. However, based on the description in the LaRC05 model [14], the resin matrix of fiber-reinforced composite laminates shows remarkable plastic hardening behavior under compressive load, as shown in Figure 5.

**Figure 6:**
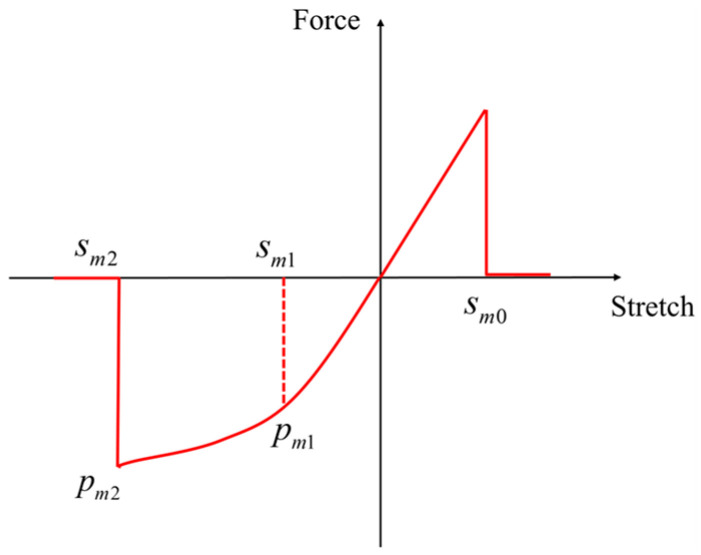
PD constitutive relation of the matrix bond.

Obviously, the existing peridynamics constitutive model of the matrix bond cannot accurately characterize the significant differences in the mechanical properties of the matrix material under tensile and compressive conditions. Therefore, based on the PMB model and the characteristics of the compression curve of the resin matrix, we propose a bond-based PD constitutive model considering the plastic hardening stage of the matrix bond, as shown in Figure 6.

##### Tensile Loading

When under tensile loading, the constitutive relation of the matrix bond is described by the PMB model [24], which is defined as

(11) $$f(\eta ,\xi )=\left\{\begin{array}{ll} c_{m}s(\eta ,\xi ) & 0\leq s(\eta ,\xi )\leq s_{m0}\\ 0 & s(\eta ,\xi )>s_{m0} \end{array}\right.,$$

where *s_m_*_0_ is related to the ultimate tensile strength of the resin matrix and defined as

(12) $$s_{m0}=\sqrt{\frac{10G_{m}}{c_{m}\pi \delta^{5}}},$$

where *G_m_* is the critical energy release rate of the matrix material.

##### Compressive Loading

In the case of matrix bond compression loading, the plastic hardening stage after the stretch exceeds the elastic limit should be considered. Therefore, the current yield force of the matrix bond is given in the form

(13) $$p_{m}=p_{m1}+H_{m}\sum \Delta s_{mp},$$

where *H_m_* can be called the plastic modulus of the matrix bond, which is given by

(14) $$H_{m}=\frac{p_{m1}-p_{m2}}{s_{m1}-s_{m2}},$$

in which Δ*s_mp_* is the plastic stretch increment of the matrix bond during a time step, and $s_{mp}=\sum ∆s_{mp}$ denotes the accumulated plastic stretch. *s_m_*_1_ and *s_m_*_2_ represent the elastic critical stretch and the fracture critical stretch of the matrix bond under compression, respectively. The elastic and fracture ultimate forces corresponding to *s_m_*_1_ and *s_m_*_2_ are *p_m_*_1_ and *p_m_*_2_.

As the matrix bond has both elastic and plastic deformation under compression, the pairwise force of the matrix bond can be expressed as

(15) $$f=c_{m}\left(s-s_{mp}\right).$$

##### Yield Criterion

In the plastic-hardening stage of the matrix bond, note that the value of the bond force absolutely cannot exceed the current yield force; however, it can be less than the current yield force in the unloading stage. Furthermore, a function that can judge whether the bond force has reached the maximum allowable value needs to be defined. Drawing on the strategy in continuum mechanics, the yield condition of the matrix bond is defined as

(16) $$\varphi \left(f,s_{mp}\right)=f-\left(p_{m1}+H_{m}s_{mp}\right).$$

According to the consistency condition, the bond force must remain at the yield force value, taking into consideration any increase caused by hardening. Thus, we can write

(17) $$\dot{\varphi }\left(f,s_{mp}\right)=\frac{\partial \varphi }{\partial f}\dot{f}+\frac{\partial \varphi }{\partial s_{mp}}{\dot{s}}_{mp}=\dot{f}-H_{m}{\dot{s}}_{mp}=0.$$

Deriving Equation (15) with respect to time, we obtain

(18) $$\dot{f}=c_{m}\left(\dot{s}-{\dot{s}}_{mp}\right),$$

where $\dot{s}$ is the stretch rate. Using Equation (4), we have

(19) $$\dot{s}=\frac{\dot{\eta }\cdot \left(\eta +\xi \right)}{\left\|\xi \right\|\cdot \left\|\eta +\xi \right\|}.$$

Combining Equations (17) and (18), the plastic stretch rate of the matrix bond can be found in Equation (20), which is called the flow rule.

(20) $${\dot{s}}_{mp}=\frac{c_{m}}{c_{m}+H_{m}}\dot{s}.$$

#### 2.2.3. Determination of Parameters in the Model of a Lamina

In the PD model of a lamina, *c_f_*, *c_m_*, *s_m_*_1_, *s_m_*_2_, *p_m_*_1_, and *p_m_*_2_ are the PD parameters related to the mechanical properties of a lamina. Next, these parameters are determined in detail.

According to the study of Oterkus et al. [29], the bond parameters *c_f_* and *c_m_* are respectively expressed as

(21) $$c_{f}=\frac{2E_{1}\left(E_{1}-E_{2}\right)}{(E_{1}-\frac{1}{9}E_{2})\left(\sum_{q}^{Q}\xi_{qi}V_{q}\right)},$$

(22) $$c_{m}=\frac{8E_{1}E_{2}}{\left(E_{1}-\frac{1}{9}E_{2}\right)\pi t\delta^{3}},$$

where *E*_1_ and *E*_2_ are the engineering elastic constants of the lamina, and *t* is the thickness of the lamina. *ξ_qi_* represents the original length of the bond between point *i* and point *q*, *V_q_* is the volume of point *q*, and *Q* denotes the total number of fiber bonds in the horizon of point *i*.

Consider a sphere with a radius of horizon size *δ* when subjected to isotropic pressure. When the deformation reaches its elastic limit, according to the continuum mechanics, the strain energy density of a material point can be given as

(23) $$U_{CCM}=\frac{1}{2}\sigma_{e}\varepsilon_{e}=\frac{1}{2}E_{m}\varepsilon_{e}{}^{2},$$

where *E_m_* is the elastic modulus of the resin matrix; $\sigma_{e}$ and $\varepsilon_{e}$ represent the elastic critical strength and strain of the resin matrix under compression, respectively.

Under the same loading condition, the strain energy density in the form of peridynamics is written as

(24) $$U_{PD}=\frac{1}{2}\int_{\Omega }\frac{c_{m}s_{m1}{}^{2}}{2}\xi d\Omega =\frac{c_{m}\pi s_{m1}{}^{2}\delta^{4}}{4}.$$

Equating the strain energy density from the peridynamics theory and classical continuum mechanics, the elastic critical stretch *s_m_*_1_ can be derived as

(25) $$s_{m1}=\sqrt{\frac{2E_{m}\varepsilon_{e}{}^{2}}{c_{m}\pi \delta^{4}}}.$$

The elastic ultimate force *p_m_*_1_ corresponding to *s_m_*_1_ is

(26) $$p_{m1}=c_{m}s_{m1}.$$

It is assumed that the ratio of *p_m_*_2_ to *p_m_*_1_ is equal to the ratio of $\sigma_{f}$ to $\sigma_{e}$ [43] and expressed as

(27) $$\frac{p_{m2}}{p_{m1}}=\frac{\sigma_{f}}{\sigma_{e}},$$

where $\sigma_{f}$ is the fracture-critical strength of the resin matrix under compression.

Hence, the fracture ultimate force *p_m_*_2_ can be derived as

(28) $$p_{m2}=\frac{\sigma_{f}}{\sigma_{e}}p_{m1}.$$

Similar to the derivation of *p_m_*_2_, the fracture critical stretch *s_m_*_2_ of the matrix bond is written as

(29) $$s_{m2}=\frac{\varepsilon_{f}}{\varepsilon_{e}}s_{m1},$$

where $\varepsilon_{f}$ is the fracture-critical strain of the resin matrix under compression.

### 2.3. PD Modeling of Laminates

**Figure 7:**
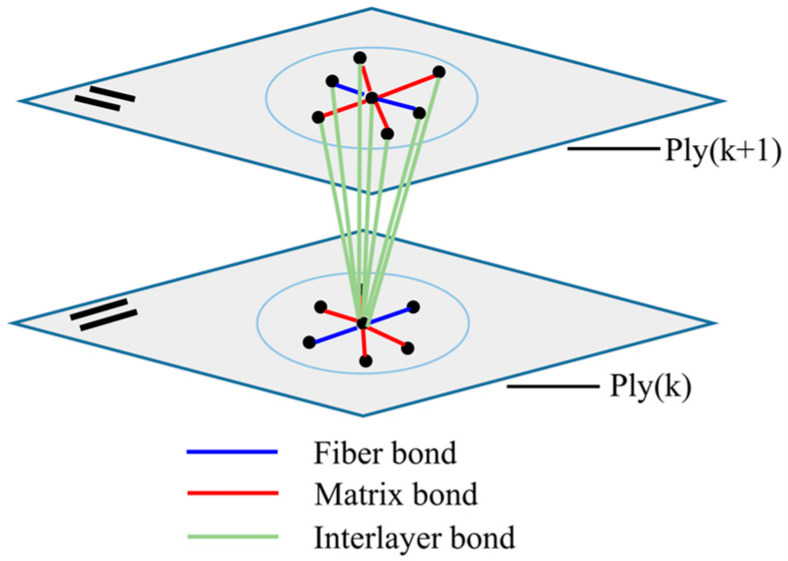
PD model of a composite laminate.

Composite laminates are composed of multiple layers of laminas with various fiber directions. There is a resin-rich layer between adjacent layers, where fracture and delamination often occur under external loading. Therefore, the mechanical behaviors of the thickness direction must be taken into account when modeling the composite laminates. In order to properly characterize this interlayer interaction, a new PD bond, called the interlayer bond with the PD material parameter *c_i_*, is defined. As shown in Figure 7, the interlayer bond only exists between the two adjacent layers, which means that the material points in the Ply(k) cannot interact with the material points in the Ply(k + 2) and Ply(k − 2). The fracture of the interlayer bond can represent the deformation behavior of a laminate in the thickness direction.

#### 2.3.1. PD Model of the Interlayer Bond

For a composite laminate, the mechanical properties of the resin-rich layer are exactly the same as those of the resin matrix. Hence, the construction process of the peridynamic constitutive model of the interlayer bond is similar to that of the matrix bond. The difference is that the parameters of the interlayer bond are *c_i_*, *s_i_*_1_, *s_i_*_2_, *p_i_*_1_, and *p_i_*_2_, while that of the matrix bond are *c_m_*, *s_m_*_1_, *s_m_*_2_, *p_m_*_1_, and *p_m_*_2_. The PD constitutive model of the interlayer bond will be briefly introduced.

##### Tensile Loading

When under tensile loading, the PD constitutive relation of the interlayer bond is defined as

(30) $$f(\eta ,\xi )=\left\{\begin{array}{ll} c_{i}s(\eta ,\xi ) & 0\leq s(\eta ,\xi )\leq s_{i0}\\ 0 & s(\eta ,\xi )>s_{i0} \end{array}\right.,$$

where *s_i0_* is the critical stretch of the interlayer bond and can be expressed as

(31) $$s_{i0}=\sqrt{\frac{10G_{m}}{c_{i}\pi \delta^{5}}}.$$

##### Compressive Loading

In the case of interlayer bond compression loading, the current yield force can be written as

(32) $$p_{i}=p_{i1}+H_{i}\sum \Delta s_{ip},$$

in which *H_i_* is called the plastic modulus of the interlayer bond. The symbol Δ*s_ip_* denotes the plastic deformation increment of the interlayer bond during a time step, and $s_{ip}=\sum ∆s_{ip}$ denotes the accumulated plastic deformation. *s_i_*_1_ and *s_i_*_2_ represent the elastic critical stretch and the fracture-critical stretch of the interlayer bond under compression, respectively. The elastic and fracture ultimate forces corresponding to *s_i_*_1_ and *s_i_*_2_ are *p_i_*_1_ and *p_i_*_2_.

The pairwise force and consistency condition of the interlayer bond under compressive load are given by

(33) $$f=c_{i}\left(s-s_{ip}\right),$$

(34) $$\dot{\varphi }\left(f,s_{ip}\right)=\frac{\partial \varphi }{\partial f}\dot{f}+\frac{\partial \varphi }{\partial s_{ip}}{\dot{s}}_{ip}=\dot{f}-H_{i}{\dot{s}}_{ip}=0.$$

#### 2.3.2. Determination of Parameters of the Interlayer Bond

The interlayer bond parameter *c_i_* can be derived in the form [34]

(35) $$c_{i}=\frac{E_{m}A}{\sum_{i}\sum_{j}\cos^{3}\alpha_{ij}V_{j}V_{i}},$$

where *E_m_* denotes the elastic modulus of the resin matrix, and *A* is the area of the lamina. The symbol $\alpha_{ij}$ denotes the initial angle formed by the interlayer bond between points *i* and *j* and the direction of the thickness of the laminate. *V_i_* and *V_j_* are the volumes of the material points *i* and *j*, respectively.

Similar to the matrix bond, the parameters *s_i_*_1_, *s_i_*_2_, *p_i_*_1_, and *p_i_*_2_ in the interlayer bond constitutive model are given as follows

(36) $$s_{i1}=\sqrt{\frac{2E_{m}\varepsilon_{e}{}^{2}}{c_{i}\pi \delta^{4}}},$$

(37) $$p_{i1}=c_{i}s_{i1},$$

(38) $$p_{i2}=\frac{\sigma_{f}}{\sigma_{e}}p_{i1},$$

(39) $$s_{i2}=\frac{\varepsilon_{f}}{\varepsilon_{e}}s_{i1}.$$

## 3. Numerical Implementation

### 3.1. Solving Method

The peridynamics equation of motion adopts the differential-integral form, which makes it difficult to obtain the analytical solution in general, but its numerical solution can be obtained by the numerical integration technique. By employing the meshless point collocation method, the computational object can be equably discretized into finite material points with their respective volumes. Then, the equation of motion in integral form can be replaced by a finite summation form and expressed as

(40) $$\rho_{i}{\ddot{u}}_{i}^{n}=\sum_{p}f\left(u_{p}^{n}-u_{i}^{n},x_{p}-x_{i}\right)V_{p}+b_{i}^{n},$$

where **f** refers to the pairwise force, *n* denotes the time step number; the subscripts are the node numbers, *V_p_* is the volume of the node *p*, and

(41) $$u_{i}^{n}=u\left(x_{i},t^{n}\right).$$

In addition, Equation (40) can be solved using the explicit central-difference technique, and the acceleration can be expressed in the following form

(42) $${\ddot{u}}_{i}^{n}=\frac{u_{i}^{n+1}-2u_{i}^{n}+u_{i}^{n-1}}{\Delta t^{2}}.$$

Furthermore, the calculation of the displacement of material point *i* at the next time step (*n* + 1) is expressed as

(43) $$u_{i}^{n+1}=\frac{\Delta t^{2}}{\rho }\left[\sum_{p}f\left(u_{p}^{n}-u_{i}^{n},x_{p}-x_{i}\right)V_{p}+b_{i}^{n}\right]+2u_{i}^{n}-u_{i}^{n-1},$$

where Δ*t* is the time step that needs to satisfy the stability condition [10]:

(44) $$\Delta t<\sqrt{\frac{2\rho }{\sum_{p=1}^{N}\frac{c}{\left\|x_{p}-x_{i}\right\|}V_{p}}},$$

where *N* is the total number of material points located in the horizon of the material point **x***_i_*.

### 3.2. Pairwise Force Updating Algorithm

For the fiber bond, the characterization of pairwise force is simple due to the linear elasticity PD constitutive relation. However, for the matrix bond and interlayer bond, the calculation of pairwise forces is more complicated because it is necessary to consider how to deal with the plastic deformation of these two types of bonds under compression loading. Drawing on the return-mapping algorithm, we propose the pairwise-force-updating algorithm to solve the real pairwise force of the matrix bond or interlayer bond in the plastic hardening stage. In the following, the matrix bond is taken as an example to introduce this algorithm in more detail.

The algorithm adopts the incremental form, which allows for consistency with the yield condition within every time step Δ*t*. The relative deformation of the matrix bond in a time step Δ*t* is expressed as

(45) $$s_{n+1}=\frac{l_{n+1}-l_{n}}{l_{n}},$$

where the subscripts *n* and *n* + 1 represent time *t* and time *t* + Δ*t,* respectively, while *l_n_* and *l_n_*_+1_ correspond to the length of the matrix bond at times *t* and *t* + Δ*t*.

Assuming that *s_n_*_+1_ is purely elastic, then the pairwise force of the matrix bond at time *t* + Δ*t* can be expressed as

(46) $$f_{n+1}^{tr}=f_{n}+c_{m}\cdot s_{n+1},$$

Here, the superscript *tr* indicates that the pairwise force in Equation (46) is not necessarily the real pairwise force of the bond because of the assumption of purely elastic deformation. However, if *s_n_*_+1_ > 0, this indicates that the bond during the time increment Δ*t* produces tensile deformation; $f_{n+1}^{tr}$ is the real pairwise force of the matrix bond.

Whether the plastic deformation of the bond occurs during the time increment Δ*t* needs to be determined by substituting $f_{n+1}^{tr}$ into the yield condition. If $f_{n+1}^{tr}$ satisfies $\varphi \left(f_{n+1}^{tr},s_{mp}\right)<0$, then the assumption of pure elasticity is correct; $f_{n+1}^{tr}$ is the pairwise force at time *t* + Δ*t*. If not, this indicates that the matrix bond has plastic deformation and $f_{n+1}^{tr}$ needs to be updated to obtain the true pairwise force. The specific calculation process is as below.

Based on Equation (20), the plastic stretch increment Δ*s_mp_* accumulated in a time step Δ*t* is solved by the following equation

(47) $$\Delta s_{mp}=\frac{c_{m}}{c_{m}+H_{m}}\dot{s}\cdot \Delta t.$$

Subsequently, the accumulated plastic stretch Δ*s_mp_* of the matrix bond at time *t* + Δ*t* can be calculated as

(48) $$s_{mp}=s_{mp}+\Delta s_{mp}.$$

Then the current yield force *p_m_* of the matrix bond at time *t* + Δ*t* is given by

(49) $$p_{m}=p_{m1}+H_{m}\Delta s_{mp}.$$

Next, $f_{n+1}^{tr}$ is updated iteratively as follows

(50) $$f_{n+1}^{\left(1\right)}=f_{n+1}^{tr},$$

(51) $$f_{n+1}^{\left(k+1\right)}=f_{n+1}^{\left(k\right)}-c_{m}\cdot \Delta s_{mp}.$$

Until $f_{n+1}^{\left(k+1\right)}$ satisfies the yield condition $\varphi \left(f_{n+1}^{\left(k+1\right)},s_{mp}\right)<0$, the true pairwise force of the matrix bond is

(52) $$f_{n+1}=f_{n+1}^{\left(k+1\right)}.$$

**Figure 8:**
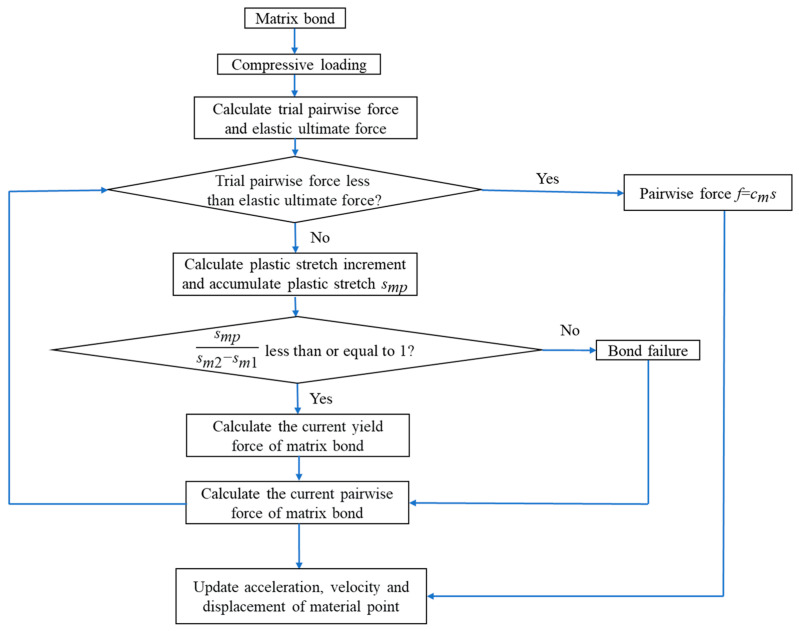
Flowchart of the pairwise force updating algorithm.

To make the algorithm presented above more intuitive and understandable, the corresponding flowchart is given, as shown in Figure 8.

## 4. Numerical Simulations

In this section, we will analyze the convergence of the PD simulation results, verify the correctness of the PD model, and investigate the impact-damage behavior and impact resistance of laminated composites with different impact velocities and layup configurations based on the established PD model. First of all, the influence of the value of the horizon on the convergence of the PD numerical simulation results was analyzed. Next, ballistic impact tests were carried out to observe the impact damage of composite laminates and compared with the simulation results. Then, a numerical example was presented to study the total process of damage evolution of composite laminates under impact loading, including damage initiation and propagation. The damage characteristics of composite laminates subjected to different impact velocities were compared, and the damage mechanism was analyzed in detail. Finally, the influence of different stacking sequences on the impact resistance of laminated composites was studied.

### 4.1. Convergence Analysis

In the study of material damage and fracture by PD theory, the reasonable selection of horizon size is crucial to ensure the convergence of results. Therefore, a simulation test of steel ball frontal impact was presented, adopting the proposed PD model, to research the *m*-convergence.

**Figure 9:**
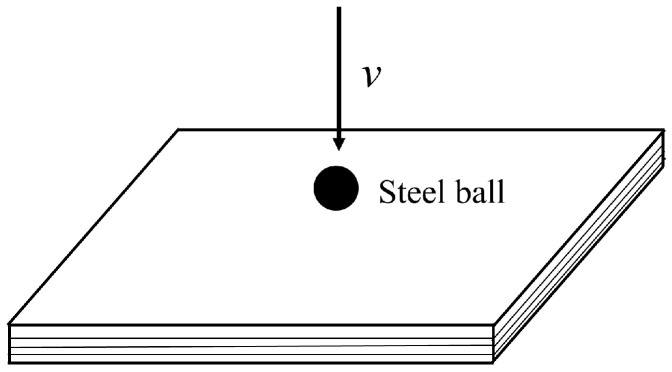
Geometric model of the laminate subjected to steel ball impact loading.

**Figure 10:**
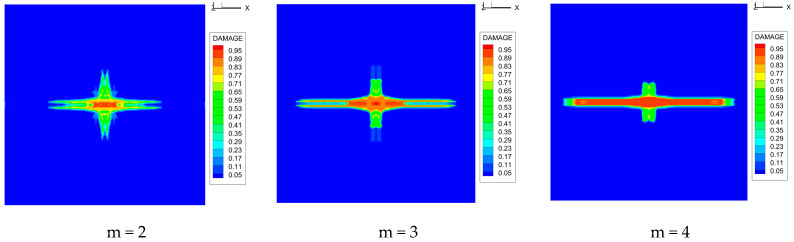
Damage pattern of composite laminate (front surface).

**Figure 11:**
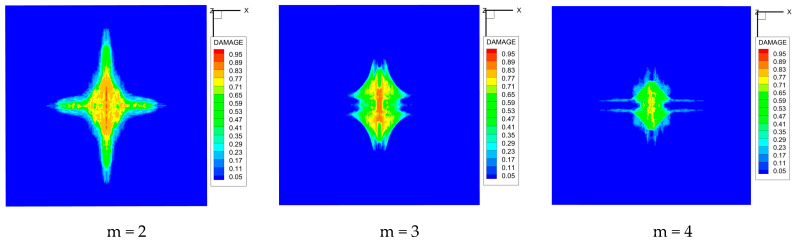
Damage pattern of composite laminate (rear surface).

As shown in Figure 9, the size of the Kevlar49/epoxy composite laminate was 100 mm × 100 mm × 6 mm. It consisted of twelve plies of laminas with a layup ${\left[0^{o}/90^{o}\right]}_{6}$ and a thickness of 0.5 mm. The material density was 1380 kg/m^3^, and the engineering constants were *E*_1_ = 78 GPa, *E*_2_ = 5.5 GPa, and $\nu_{12}=0.33$. The projectile was a steel ball with a diameter of 6 mm and a mass of 0.84 g, and the impact velocity in this test was 300 m/s. The ratio of horizon *δ* to particle spacing Δ*x* can be defined as *m*; that is, m=δ/Δx. In the *m*-convergence, the particle spacing Δ*x* is fixed at 0.5 mm. Three different *m* values (*m* = 2, 3, 4) were selected, and the corresponding horizons were *δ* = 1Δ*x*, 2Δ*x*, and 3Δ*x*. The damage conditions of laminate corresponding to different sizes of the horizon are shown in Figure 10 and Figure 11.

The simulation results indicate that the damage results are more divergent when *m* = 2, and the laminate damage becomes more convergent with the increase in the horizon size. For a fixed particle spacing Δ*x*, when *m* increases, the damage characteristics of composite laminate are more distinctive and more in line with the failure modes summarized in the literature [44]. The laminate damage can be captured when *m* is equal to 3 or 4. Therefore, *m* = 3 will be used in all the remaining simulation examples, considering the computational efficiency.

### 4.2. Verification

**Figure 12:**
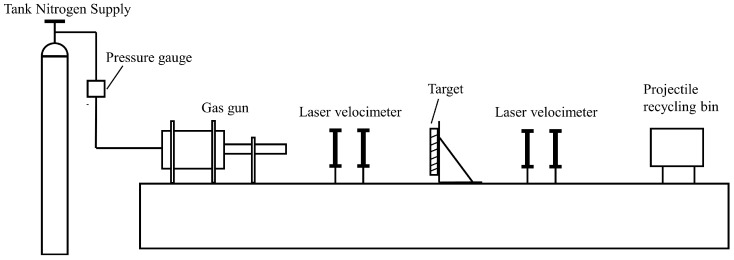
Schematic illustration of high-speed impact device.

For comparison with the simulation results and to prove the proposed PD model’s ability to capture the impact–damage process and features of composite materials, we conducted ballistic impact tests of composite laminates. Kevlar49/epoxy composite laminates with sizes of 100 mm × 100 mm × 6 mm were used in the study. Each laminate consisted of twelve plies of laminas with a layup ${\left[0^{o}/90^{o}\right]}_{6}$ and a thickness of 0.5 mm. The material density was 1380 kg/m^3^, and the engineering constants were *E*_1_ = 78 GPa, *E*_2_ = 5.5 GPa, and $\nu_{12}=0.33$. The ballistic tests were carried out on a high-speed impact device, as shown in Figure 12.

The projectile employed in the present work was a steel ball with a diameter and mass of 6 mm and 0.84 g, respectively, and initial impact velocities were 100 m/s, 200 m/s, and 300 m/s. In all tests, the target was fixedly placed 1 m away from the nuzzle end of the gun. A laser velocimetry system was used to record the impact and residual velocities of the projectile in each test. The damage results of laminates under different impact speeds are shown in Table 1. Note that the non-English parts in Table 1 mean “steel ball”.

**Figure 13:**
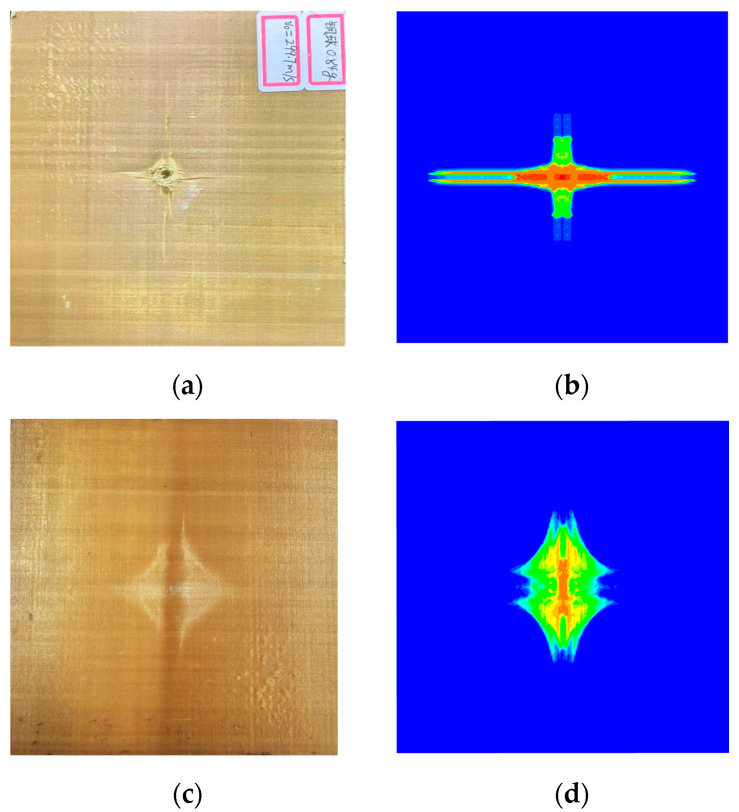
(**a**) Experimental result of the front surface; (**b**) PD simulation result of the front surface; (**c**) Experimental result of the rear surface; (**d**) PD simulation result of the rear surface.

The experimental results of laminate damage were compared with the PD numerical simulation results, as shown in Figure 13. Note that the non-English part in Figure 13 means “steel ball”. The simulation results accurately reflect the damage behaviors and characteristics of the laminate and are in high agreement with the experimental results, which proves the validity and accuracy of the established PD model with matrix plasticity for composite materials.

### 4.3. Initiation and Propagation of Damage

A rectangular composite laminate with the size of 100 × 100 × 6 mm^3^ is shown in Figure 9, with a density of 1380 kg/m^3^. This laminate has 12 plies with layup [0/90]_6_. Each unidirectional lamina is composed of Kevlar49/epoxy with mechanical properties of *E*_1_ = 78 GPa, *E*_2_ = 5.5 GPa, and $\nu_{12}=0.33$. The steel ball is considered to be a rigid body with a diameter of 6 mm and a mass of 0.84 g. The impact velocity of the steel ball is 300 m/s.

As a target model, the laminate was divided into 200 × 200 × 12 material points with a particle spacing of 0.5 mm. The horizon size of the material point is 1.5 mm, which is three times the size of the particle spacing. The time step was chosen as $t=1{.0\;\times \;10}^{-9}\;s$, which can satisfy the stability condition and ensure high computational efficiency. The time evolution of damage and fracture on the front surface, rear surface, and cross-section of the composite laminate is illustrated in Table 2, Table 3 and Table 4.

According to the damage evolution of the front and rear surfaces, it can be observed that the laminate damage first occurred at the center of the front surface, as the compression stress wave generated by the steel ball impacted the target plate. Until $t=2{.0\;\times \;10}^{-5}\;s$, the tensile wave reflected from the back side caused a small, damaged area on the back side of the laminate. Next, as the steel ball continued to penetrate the laminate, the damage on the front and back sides propagates and grows. Finally, when $t=5{.0\;\times \;10}^{-5}\;s$, the front surface of the laminate formed a cross-damaged zone along the fiber direction, while the damage zone on the back surface expanded into a diamond shape with the fiber direction as the long axis.

During the damage evolution of laminate, we noticed an obvious characteristic: the damage on the front and rear surfaces of laminate always propagated along the fiber direction during the impact process, showing significant anisotropy and consistent with the conclusion in the literature [45]. This is probably because the stress wave in the laminate travels faster along the fiber direction than along the other directions [44]. Another possible reason is that the fracture strength of the fiber is much higher than that of the resin matrix. When the fibers break, the cracks in the matrix rapidly propagate along the fiber orientation. In addition, the damaged area on the back side of the laminate is greater than that on the front side due to the tensile wave reflected by the free surface of the laminate [46].

According to the change in cross-sectional damage to the laminate over time, the process and characteristics of delamination can be observed. At the early stage, when the steel ball impacts the target plate, the center of the impact surface is penetrated by the steel ball to a certain extent, and delamination damage begins to appear. At $t=2{.0\;\times \;10}^{-5}\;s$, the further cracking of the resin-rich layer deepens the degree of delamination. This initial impact damage generally occurs in the vicinity of the impact surface and is related to the high shear stress caused by the penetration of steel balls on the laminate [47,48]. Under high shear stress, the transverse shear cracks generated by the resin matrix continuously collect at the interface between two laminas, which leads to the delamination of the composite laminate [47,48]. When $t=3{.0\;\times \;10}^{-5}\;s$ and $t=4{.0\;\times \;10}^{-5}\;s$, the transverse shear failure effect of the matrix diminishes as the degree of penetration increases. Therefore, although the delamination near the impact surface is still expanding, the rate of expansion is significantly lower than that which occurs in the early stage of penetration. Conversely, the delamination of the laminate backside propagates faster than in the early stages of penetration. This is because the tensile action generated by the bending deformation is continuously intensified along the thickness direction of the laminate [47,48].

Through the above work, we can obtain the damage on the front and rear surfaces and the delamination in the thickness direction over time using the proposed PD model, which will provide an important contribution to the in-depth understanding and investigation of the behavioral characteristics and mechanical mechanisms of impact-induced damage to composite materials.

### 4.4. Damage Pattern under Different Impact Velocities

Based on the proposed PD model, the dynamic mechanical behavior of composite laminates under different impact velocities will subsequently be investigated. The size and properties of the composite laminate model are the same as those in Section 4.2. The impact velocities of the steel balls are 100 m/s, 200 m/s, 300 m/s, 500 m/s, and 700 m/s. The damage results of the laminates under different impact velocities are presented in Table 5.

The analysis of Table 5 shows that with the continuous increase in impact velocity, the damage to the laminate is also intensified. The reason for this tendency is probably that the increase in impact velocity continuously deepens the propagation and superposition of impact waves inside the target plate. Under the lower impact velocities of 100 m/s and 200 m/s, the laminate is penetrated by the steel ball to a certain extent, and the impact damage is mainly distributed on the front surface. The impact damage on the rear surface is not obvious, and there is no damage on the rear surface when subjected to the impact velocity of 100 m/s.

However, when subjected to higher impact velocities like 300 m/s, 500 m/s, and 700 m/s, the damage to the laminates is significant at both the front and rear surfaces and far more dramatic than that under the lower impact velocities. It was observed that at 500 m/s and 700 m/s impact velocities, the damage along the fiber direction on the front surface was extended to the edge of the laminates. A strip of delamination with a width equal to the diameter of the steel ball was observed on the rear surface of the laminate, extending along the fiber direction of $90^{o}$ to the boundary of the target plate, which is in high agreement with the conclusion in the literature [49]. This damage pattern is determined by the fiber layup direction of the laminated composite. Each layer of fibers in the laminate is unidirectional and connected by resin bonding, which has a much lower strength than the fibers and is easy to fracture. Under the higher impact velocities, when the fibers on the back side of the laminate break under tension, the cracks in the resin matrix will rapidly propagate in the direction of the fibers, causing the resin bonding to disappear and forming a strip of delamination.

### 4.5. Impact Resistance with Different Stacking Sequences

In this section, the impact resistance of composite laminates with different stacking sequences is studied. The layup configurations of laminates are ${\left[0^{o}\right]}_{12}$, ${\left[90^{o}\right]}_{12}$, ${\left[0^{o}/90^{o}\right]}_{6}$ and ${\left[45^{o}/0^{o}/-45^{o}/90^{o}\right]}_{3}$ and ${\left[{\left(0^{o},90^{o}\right)}_{1}\right]}_{12}$ woven-fabric stacking. The single lamina had the same size and engineering constants as in Section 4.2. The projectile used was still a steel ball with an impact speed of 300 m/s. The simulation results of the laminated composites with different layup configurations are presented in Table 6.

It can be seen that layup configuration has a significant influence on the impact resistance of composite laminate. The damage on the front and rear surfaces of ${\left[0^{o}\right]}_{12}$ and ${\left[90^{o}\right]}_{12}$ unidirectional laminates extended along the fiber direction to the edge of the laminates, forming a strip of delamination with a width close to that of the diameter of the steel ball. By comparing the ${\left[0^{o}/90^{o}\right]}_{6}$ laminate with the ${\left[0^{o}\right]}_{12}$ and ${\left[90^{o}\right]}_{12}$ unidirectional laminates, the damage on the front surface of ${\left[0^{o}/90^{o}\right]}_{6}$ the laminate is shown to extend along the fiber direction but does not form a strip of delamination like that of unidirectional laminates. In addition, the damaged area on the rear surface of ${\left[0^{o}/90^{o}\right]}_{6}$ laminate expands to a diamond shape instead of a strip of delamination, and the damage degree is smaller than that of unidirectional laminates. Therefore, the impact resistance of ${\left[0^{o}/90^{o}\right]}_{6}$ laminate is better than that of ${\left[0^{o}\right]}_{12}$ and ${\left[90^{o}\right]}_{12}$ unidirectional laminates.

Compared with ${\left[0^{o}\right]}_{12}$, ${\left[90^{o}\right]}_{12}$, and ${\left[0^{o}/90^{o}\right]}_{6}$ laminates, the damage degree of ${\left[{\left(0^{o},90^{o}\right)}_{1}\right]}_{12}$ woven-fabric laminate is smaller and has better impact resistance. This is because the fibers of the same layer of the woven fabric laminate have two directions, meaning that the anisotropy characteristics of the woven-fabric laminate are not obvious; therefore, the in-plane deformation and force are more uniform [50]. The steel ball can only penetrate through the target plate when all the interconnecting fibers fracture.

The damaged area on the front and rear surfaces of the ${\left[45^{o}/0^{o}/-45^{o}/90^{o}\right]}_{3}$ laminate is irregular, and the damage is less serious than that of ${\left[0^{o}\right]}_{12}$, ${\left[90^{o}\right]}_{12}$, and ${\left[0^{o}/90^{o}\right]}_{6}$ laminates. These simulation results show that increasing the layup orientation can effectively improve the impact resistance of composite laminates, which is consistent with the conclusion in the literature [51]. A reasonable explanation is that the $\pm 45^{o}$ layering of fibers makes the stress transfer in the laminate more uniform, thus improving the strength and impact resistance of the ${\left[45^{o}/0^{o}/-45^{o}/90^{o}\right]}_{3}$ laminate.

## 5. Conclusions

In this study, a bond-based peridynamic model that considers the plastic hardening of resin matrix for fiber-reinforced composite materials was developed based on the PMB model and the LaRC05 failure model. A pairwise-force-updating algorithm was put forward to calculate the true bond forces of the matrix bond and interlayer bond with the plastic-hardening behavior. This PD model was employed to investigate the damage evolution and failure characteristics of composite laminates subjected to impact loading. Ballistic impact tests of composite laminates were conducted to observe the phenomenon and characteristics of impact damage. The simulation results, based on the model established in this research, match the experimental results very well. In addition, this PD model was adopted to investigate the impact-induced damage features of composite laminates under different impact velocities and with different stacking sequences. Some conclusions are given as follows.

1. The developed bond-based peridynamic model can accurately describe the impact-induced damage behavior and evolution of fiber-reinforced composite materials. The PD simulation results for impact damage showed a good match with the experimental phenomena.
2. The damage to composite laminates under impact loading is distributed and propagated along the fiber orientation, which shows significant anisotropy.
3. The damage to composite laminates will become more and more severe with the increase in impact velocity. Compared with a low-impact velocity, the damage to the rear surface of laminates is more serious under high-impact velocity, and a strip of delamination is formed along the fiber orientation.
4. The stacking sequence has a distinct effect on the impact resistance of composite laminates under the same impact velocity. For the angle ply laminate, increasing the fiber layup orientation will significantly improve its impact resistance.

## Figures and Tables

**Table 1:** The damage to Kevlar49/epoxy composite laminates under ballistic impact.

Projectile Velocity	100 m/s	200 m/s	300 m/s
Front surface	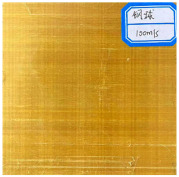	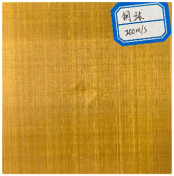	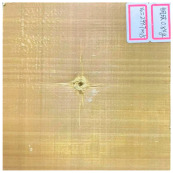
Rear surface	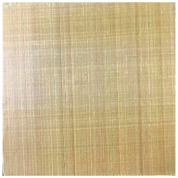	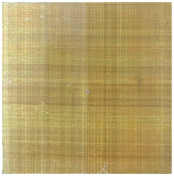	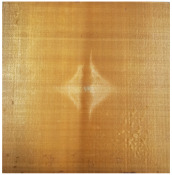

**Table 2:** Laminate damage evolution process with time (front surface).

Time	Tensile Damage	Compressive Damage	Total Damage
$t=1{.0\;\times \;10}^{-5}\;s$	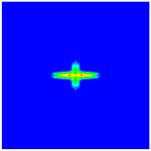	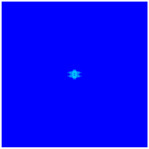	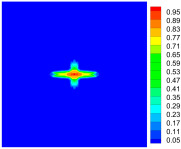
$t=2{.0\;\times \;10}^{-5}\;s$	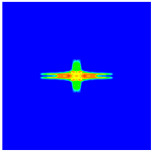	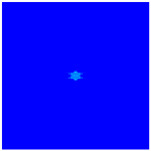	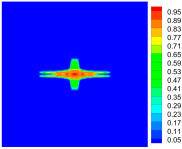
$t=3{.0\;\times \;10}^{-5}\;s$	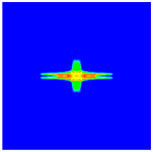	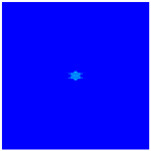	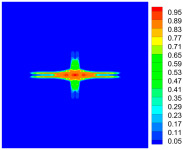
$t=4{.0\;\times \;10}^{-5}\;s$	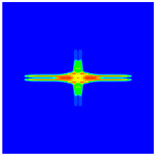	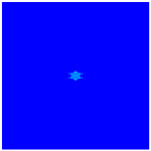	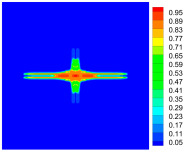
$t=5{.0\;\times \;10}^{-5}\;s$	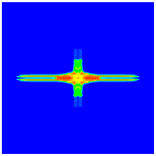	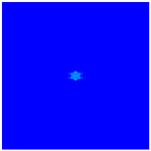	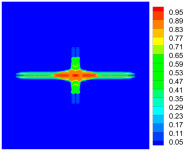

**Table 3:** Laminate damage evolution process with time (rear surface).

Time	Tensile Damage	Compressive Damage	Total Damage
$t=1{.0\;\times \;10}^{-5}\;s$	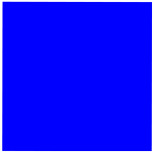	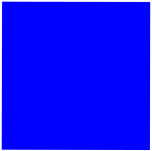	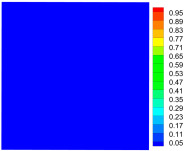
$t=2{.0\;\times \;10}^{-5}\;s$	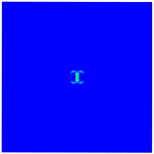	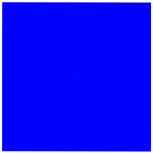	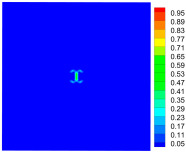
$t=3{.0\;\times \;10}^{-5}\;s$	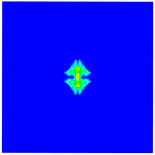	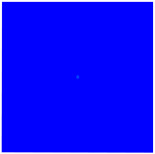	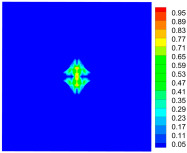
$t=4{.0\;\times \;10}^{-5}\;s$	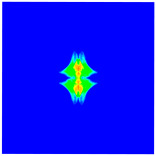	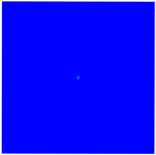	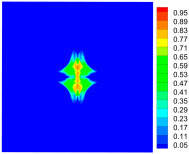
$t=5{.0\;\times \;10}^{-5}\;s$	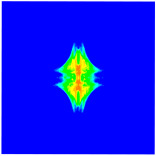	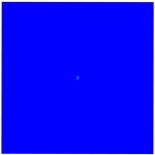	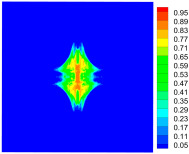

**Table 4:** Delamination damage of the cross-section.

Time	Delamination Damage
$t=1{.0\;\times \;10}^{-5}\;s$	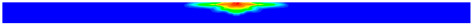
$t=2{.0\;\times \;10}^{-5}\;s$	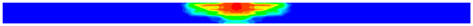
$t=3{.0\;\times \;10}^{-5}\;s$	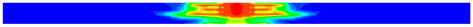
$t=4{.0\;\times \;10}^{-5}\;s$	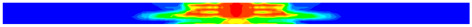
$t=5{.0\;\times \;10}^{-5\;}s$	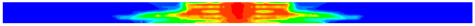

**Table 5:** Damage to the laminates subjected to different impact velocities.

Projectile Velocity	100 m/s	200 m/s	300 m/s	500 m/s	700 m/s
Front Surface	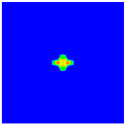	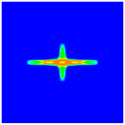	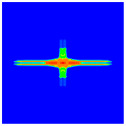	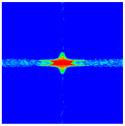	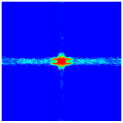
Rear Surface	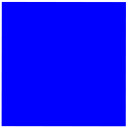	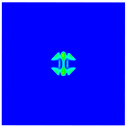	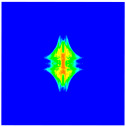	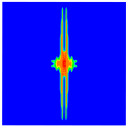	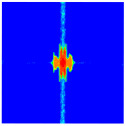

**Table 6:** Damage of the laminates with different stacking sequences.

Stacking Sequences	Front Surface	Rear Surface
${\left[0^{o}\right]}_{12}$	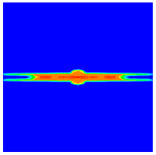	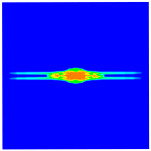
${\left[90^{o}\right]}_{12}$	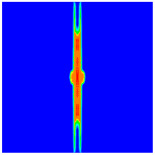	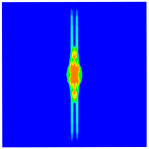
${\left[0^{o}/90^{o}\right]}_{6}$	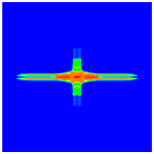	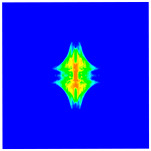
${\left[{\left(0^{o},90^{o}\right)}_{1}\right]}_{12}$	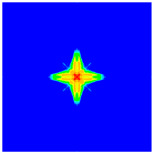	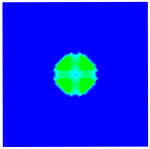
${\left[45^{o}/0^{o}/-45^{o}/90^{o}\right]}_{3}$	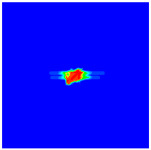	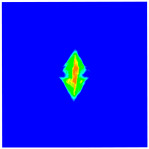

## Data Availability

The data used to support the findings of this study are available upon request from the corresponding author.
